# Unravelling the Role of LncRNA WT1-AS/miR-206/NAMPT Axis as Prognostic Biomarkers in Lung Adenocarcinoma

**DOI:** 10.3390/biom11020203

**Published:** 2021-02-02

**Authors:** Wen Li, Yu Liu, Zi Jin Li, Yi Shi, Jing Deng, Jie Bai, Liang Ma, Xiao Xi Zeng, Shan Shan Feng, Jia Li Ren, Fei Jun Luo, Duo Yan Rong, Xiao Qi Chen, Hua Qun Yin, Zhu Chen, Fu Da

**Affiliations:** 1 College of Life Sciences and Chemistry, Hunan University of Technology, Zhuzhou 412007, China; liwen@hut.edu.cn (W.L.); M18077700006@hut.edu.cn (Y.L.); M18077700011@hut.edu.cn (Y.S.); maliang@hut.edu.cn (L.M.); zengxiaoxi@hut.edu.cn (X.X.Z.); M19077700015@hut.edu.cn (S.S.F.); rongduoyan@hut.edu.cn (D.Y.R.); 2 College of Food Science and Engineering, Central South University of Forestry and Technology, Changsha 410004, China; 20183530@csuft.edu.cn (Z.J.L.); T20071464@csuft.edu.cn (J.B.); T20091483@csuft.edu.cn (J.L.R.); T20121480@csuft.edu.cn (F.J.L.); 20183520@csuft.edu.cn (X.Q.C.); 3 School of Resource Processing and Bioengineering, Central South University, Changsha 410083, China; yinhuaqun@csu.edu.cn; 4 Central Laboratory for Medical Research, Shanghai Tenth People’s Hospital, Tong Ji University School of Medicine, Shanghai 200072, China

**Keywords:** LUAD, ceRNA network, WT1-AS/miR-206/NAMPT axis, prognosis

## Abstract

Lung cancer is the world’s highest morbidity and mortality of malignant tumors, with lung adenocarcinoma (LUAD) as a major subtype. The competitive endogenous RNA (ceRNA) regulative network provides opportunities to understand the relationships among different molecules, as well as the regulative mechanisms among them in order to investigate the whole transcriptome landscape in cancer pathology. We designed this work to explore the role of a key oncogene, MYC, in the pathogenesis of LUAD, and this study aims to identify important long noncoding RNA (lncRNA)-microRNA (miRNA)- transcription factor (TF) interactions in non-small cell lung cancer (NSCLC) using a bioinformatics analysis. The Cancer Genome Atlas (TCGA) database, containing mRNA expression data of NSCLC, was used to determine the deferentially expressed genes (DEGs), and the ceRNA network was composed of WT1-AS, miR-206, and nicotinamide phosphoribosyltransferase (NAMPT) bashing on the MYC expression level. The Kaplan–Meier univariate survival analysis showed that these components may be closely related prognostic biomarkers and will become new ideas for NSCLC treatment. Moreover, the high expression of WT1-AS and NAMPT and low expression of miR-206 were associated with a shortened survival in NSCLC patients, which provided a survival advantage. In summary, the current study constructing a ceRNA-based WT1-AS/miR-206/NAMPT axis might be a novel important prognostic factor associated with the diagnosis and prognosis of LUAD.

## 1. Introduction

Lung cancer is the world’s highest morbidity and mortality of malignant tumors, a grievous menace to human health, and more than 1.8 million people died of lung cancer in 2018, according to the World Health Organization. With advances in technology and medical treatment, many diseases such as cardiovascular disease and infectious diseases have been effectively controlled or treated. However, the morbidity and mortality of lung cancer are rising year by year, which is attributed to the aggravation of environmental pollution and the lack of effective early diagnosis and treatment of lung cancer. Hence, it is necessary to explore the pathogenesis of lung cancer and find biomarkers for the early diagnosis of lung cancer [1,2,3].

Lung cancer is classified into small cell lung cancer (SCLC) and non-small cell lung cancer (NSCLC), according to the pathological features, and NSCLC accounts for about 85% of the newly diagnosed lung cancer cases. NSCLC mainly includes three pathologic types: lung adenocarcinoma (LUAD), lung squamous cell carcinoma (LUSC), and large cell lung cancer (LCLC). Adenosquamous carcinoma is an infrequent histological type of NSCLC, accounting for 0.6–2.3% of whole lung cancer samples, and it is difficult to diagnose because of adenosquamous carcinoma expressing the clinical and pathological characteristics of LUAD and LUSC. Moreover, there is a risk of misdiagnosis [4]. NSCLC has been treated in a variety of ways, including surgery, chemotherapy, targeted therapy, and immunotherapy, but also individually or in combination. Overall, nevertheless, the five-year overall survival (OS) rate for lung cancer patients was not satisfactory, at 68% in stage I patients and less than 10% in stage IVA-IVB patients. Therefore, researchers must have a deeper understanding of the mechanism of NSCLC, explore new and effective screening methods for NSCLC, and develop new antitumor drugs to improve the treatment effects of NSCLC patients and prolong their survival [2,4,5].

Long noncoding RNAs are endogenous RNA molecules greater than 200 bp in length that do not encode proteins. Recent studies have shown that lncRNA, as a competitive, endogenous RNA, can adsorption microRNAs (miRNAs) and participate in the regulation of target gene expression, thus playing a crucial role in the occurrence and development of tumors [6]. The CeRNA (competing endogenous RNAs) hypothesis reveals a new mechanism for RNAs to interact with each other. MicroRNAs (miRNA) cause gene silencing by uniting mRNAs, while ceRNA regulates the gene expression by competitively uniting miRNAs. CeRNA can bind to miRNAs through response elements to affect miRNA binding and thereby affect gene silencing caused by miRNA, which reveals the existence of an RNA and miRNA regulatory pathway and has great biological significance [7].

MYC is located on chromosome 8 q24.21 and proven to be a control gene involving cancer through the recruitment of histone acetyltransferase and combining an enhancer box sequence to control about 15% of the human gene expression [8]. Abnormal MYC genes lead to uncontrollable MYC expression in malignant tumors because of dysfunction and translocation among Burkitt lymphoma, cervical cancer, colon cancer, breast cancer, stomach cancer, and lung cancer, especially in the pathogenesis of B-cell lymphoma, which plays an important role [9,10]. Studies have shown that MYC overexpression occurs in 41% of NSCLC patients and is associated with the loss of cell differentiation events [10]. In addition to the function of the classic, MYC promotes tumorigenic immune evasion by inducing the expression of integrin-associated protein, (IAP, CD47) and programmed death-ligand (PD-L1) [11]. Casey found that the use of tumor cells and mice models, such as mice and human tumor cells, inhibits MYC, resulting in a loss of the PD-L1 mRNA and protein levels. MYC is directly linked with the PD combination of the L1 gene promoter, and the inactivation of MYC can downregulate the PD-L1 expression in mice and enhance the antitumor immune response [11].

To explore the relationship between the expression of MYC, a key oncogene, and the development of lung cancer, and to find the MYC expression-related regulatory pathways involved in the occurrence of LUAD, a ceRNA expressed network was constructed.

## 2. Materials and Method

### 2.1. Materials

TCGA (https://CancerGenome.nih.gov/), R version 3.6.0 (https://cran.rproject.org/bin/windows/base/), edgeR package, (https://bioconductor.org/packages/release/bioc/html/edgeR/html), miRcode (https://www.miRcode.org/), miRTarBase (https://mirtarbase.mbc.nctu.edu.tw/), miRDB (https://ww.mirdb.org/), TagetScan (https://targetscan.org/vert71), Survival package (https://bioconduc-tor.org/biocLite.R/), GEPIA (http://gepia.cancer-pku.cn/), GEO (https://www.ncbi.nlm.nih.gov/geo/), HPA (https://www.proteinatlas.org/), GeneCards (https://www.genecards.org/), cbioportal (https://www.cbioportal.org/), imer (http://timer.cistrome.org/), Cystoscape V3.6.0 (http://cytoscape.org/).

### 2.2. NSCLC RNA Sequencing Data Extraction

The RNA sequencing data were obtained from the The Cancer Genome Atlas (TCGA) database with 535 LUAD patients. The inclusion criteria were as follows: (1) The clinical sample of the patient was pathologically confirmed as LUAD, (2) the tumor stage and histological classification of the patients were clearly confirmed, (3) the patient was diagnosed for the first time and did not receive other radiotherapy or chemotherapy before surgery, (4) no serious lesions were found in the patient’s vital organs, and (5) the patient had no other complicated tumors. The exclusion criteria were for patient data: (1) The patient could not be diagnosed as LUAD by a pathological diagnosis, (2) there was no complete follow-up data of the patients after surgery, (3) the patient has cardiovascular disease or other infectious diseases, (4) patients with other pulmonary diseases, and (5) the miRNA data of the patient data were not matched between the mRNA and lncRNA. Finally, the expression data and clinical information of 515 patients were selected to be included in this study.

### 2.3. Differential Gene Expression Analysis Using the TCGA Database

All RNA sequencing and clinical data of LUAD were downloaded from the TCGA database using the “gdc-client.exe” tool on 16 March 2020. These data were derived from 515 LUAD tumor samples and 46 normal adjacent samples, respectively. LUAD patients (515) were divided into two groups according to the MYC expression median (MYC expression = 2541.5)—namely, the low expression group (MYC expression < 2541.5, *n* = 257) and high expression group (MYC expression > 2541.5, *n* = 258). The following used the “edgeR” package (version 3.22.0) in R software to identify differentially expressed lncRNAs (DElncRNAs) and differentially expressed miRNAs (DEmiRNAs) with the thresholds of |log2|FC|| > 1 and adjusted *p*-value < 0.01 and identify differentially expressed mRNAs (DEmRNAs). The thresholds were |long2|FC|| > 0.5 and adjusted *p*-value < 0.01.

### 2.4. Prediction of lncRNAs Targeted by miRNAs

Relevant miRNA-target data were predicted from the miRcode database, and the interaction was predicted based on the combination of differentially expressed ones between lncRNAs and miRNAs.

### 2.5. Prediction of Targeted mRNAs of miRNAs

The miPDB, miRarBase, and TargetScan databases were downloaded, and the miRNA-targeted mRNA predicted by the three databases were intersected with the differentially expressed mRNA to obtain the final target mRNA.

### 2.6. Constructing the ceRNA Network and Functional Enrichment Analysis

An interaction was established between lncRNA–miRNA and miRNA–mRNA, and lncRNA and miRNAs were obtained through the interaction of lncRNA–miRNA predicted by miRcode in the ceRNA network. The final target mRNA was the intersection between the differentially expressed mRNA and target mRNA predicted by the “database3.0” database Kyoto Encyclopedia of Genes and Genomes (KEGG) analysis; *p* < 0.05 was considered statistically significant.

### 2.7. Survival Analysis

In order to determine the DElncRNA, DemiRNA, and DemRNA in the ceRNA network, the clinical data of patients with LUAD in the TCGA were combined to perform a Kaplan–Meier univariate survival analysis. GraphPad Prism 8 was used to draw the survival curve of the DElncRNA, DemiRNA, and DemRNA in the ceRNA network, and *p* < 0.05 was considered statistically significant.

## 3. Results

### 3.1. LUAD and MYC Expression

#### 3.1.1. Identification of MYC in LUAD

**Figure 1:**
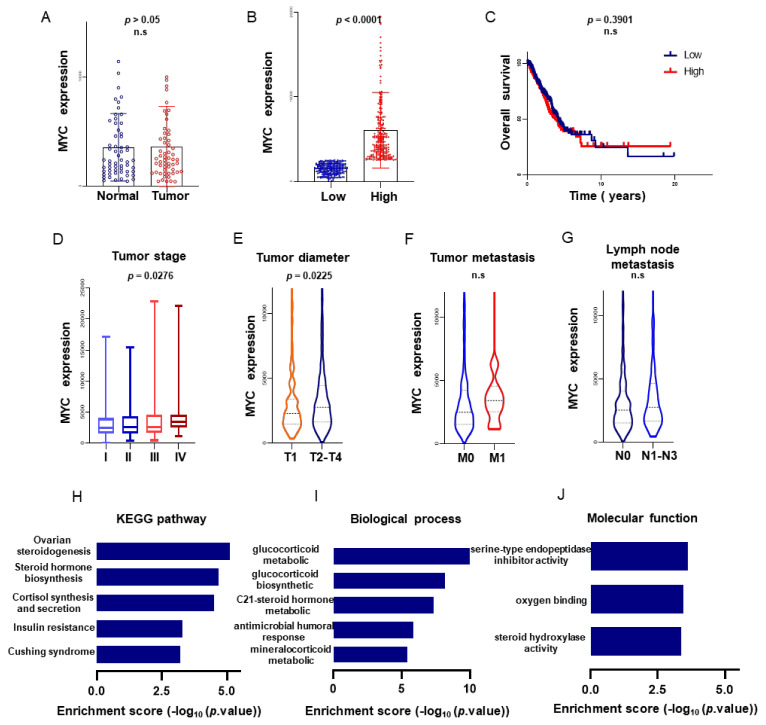
Expression of MYC in lung adenocarcinoma (LUAD). There was no significant difference in MYC expression in LUAD tissues and adjacent tissues (**A**). According to the MYC expression level, patients with LUAD were divided into high expression levels and low expression levels (**B**). There was no significant difference between the MYC expression level and patient survival time (**C**). The MYC expression level was associated with tumor stage, lymph node metastasis, tumor metastasis, and tumor diameter (**D**–**G**). The Kyoto Encyclopedia of Genes and Genomes (KEGG) analysis and gene ontology (GO) analysis of differentially expressed genes related to MYC (**H**–**J**).

The MYC gene expression dataset and clinical information of 515 LUAD patients were downloaded from The Cancer Genome Atlas (TCGA) database, 59 of whom had paracancer tissues corresponding to their tumor sites. The MYC expression in the LUAD samples was not significantly different than that in the adjacent tissues, calculated by the relative standard deviation (*p* > 0.05, Figure 1A). And MYC gene Immunohistochemistry in patients with lung adenocarcinoma) was shown in Figure S1.

In order to analyze whether the MYC expression level has an impact on patient survival, the correlation between MYC expression and patient survival time was shown. The MYC gene expression level of 515 LUAD patients was divided into a high expression group (*n* = 257) and a low expression group (*n* = 258; Figure 1B) according to the median level, and the corresponding clinical information was combined to draw the survival curve (Figure 1C). However, the analysis showed no significant association between the MYC expression and patient survival time (*p* = 0.309). 

The correlation was shown in Table 1 and Figure 1 between the MYC expression and patient clinical factors, which were analyzed to find out whether the MYC expression level has an impact on the clinical characteristics. It was found that the MYC expression level in patients with LUAD included in this study was positively correlated with the smoking history of patients, tumor stage (*p* = 0.028; Figure 1D), and tumor diameter (*p* = 0.023; Figure 1E and Table 1). However, the analysis showed no association between the MYC expression in patient age, sex, tumor metastasis (*p* = 0.025; Figure 1F), and lymph node metastasis (*p* > 0.05; Figure 1G). Tumor stage, tumor, tumor node, and tumor diameter can be used as independent prognostic factors to affect the survival of patients (Table 1). 

#### 3.1.2. Kyoto Encyclopedia of Genes and Genomes (KEGG) Pathway Analysis

In order to clarify the relationship between normal and tumor cells, we grouped other genes according to how much MYC was expressed and obtained 213 differentially expressed genes—among which, 141 (66.20%) genes were upregulated, and 72 (33.80%) genes were downregulated (log2|FC| = 2, *p* < 0.05). The KEGG analysis of these differentially expressed genes revealed many regulatory pathways associated with cancer cells, such as ovarian steroidogenesis, steroid hormone biosynthesis, cortisol synthesis, and the secretion hormone synthesis pathway (Figure 1H).

#### 3.1.3. Gene Ontology (GO) Analysis

Functional bias was detected in 213 differentially expressed transcripts based on a GO analysis. According to the GO analysis results, these genes were divided into 57 GO sequences, the most abundant of which included the glucocorticoid metabolic process, glucocorticoid biosynthetic process, C21 hormone metabolic process, response to mineralocorticoid, and epidermis development (Figure 1I). The molecular functional analysis showed that the enrichment of differentially expressed genes was correlated with enzyme activity and oxygen binding (Figure 1J). 

### 3.2. Differential RNA in LUAD

#### 3.2.1. Identification of DElncRNA in LUAD

**Figure 2:**
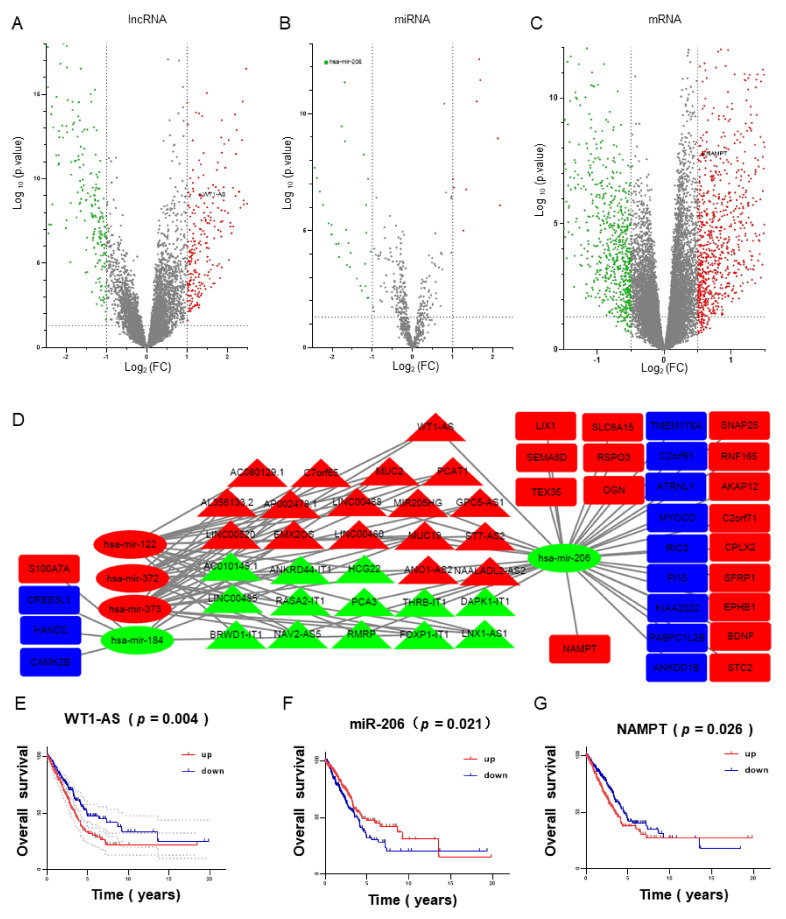

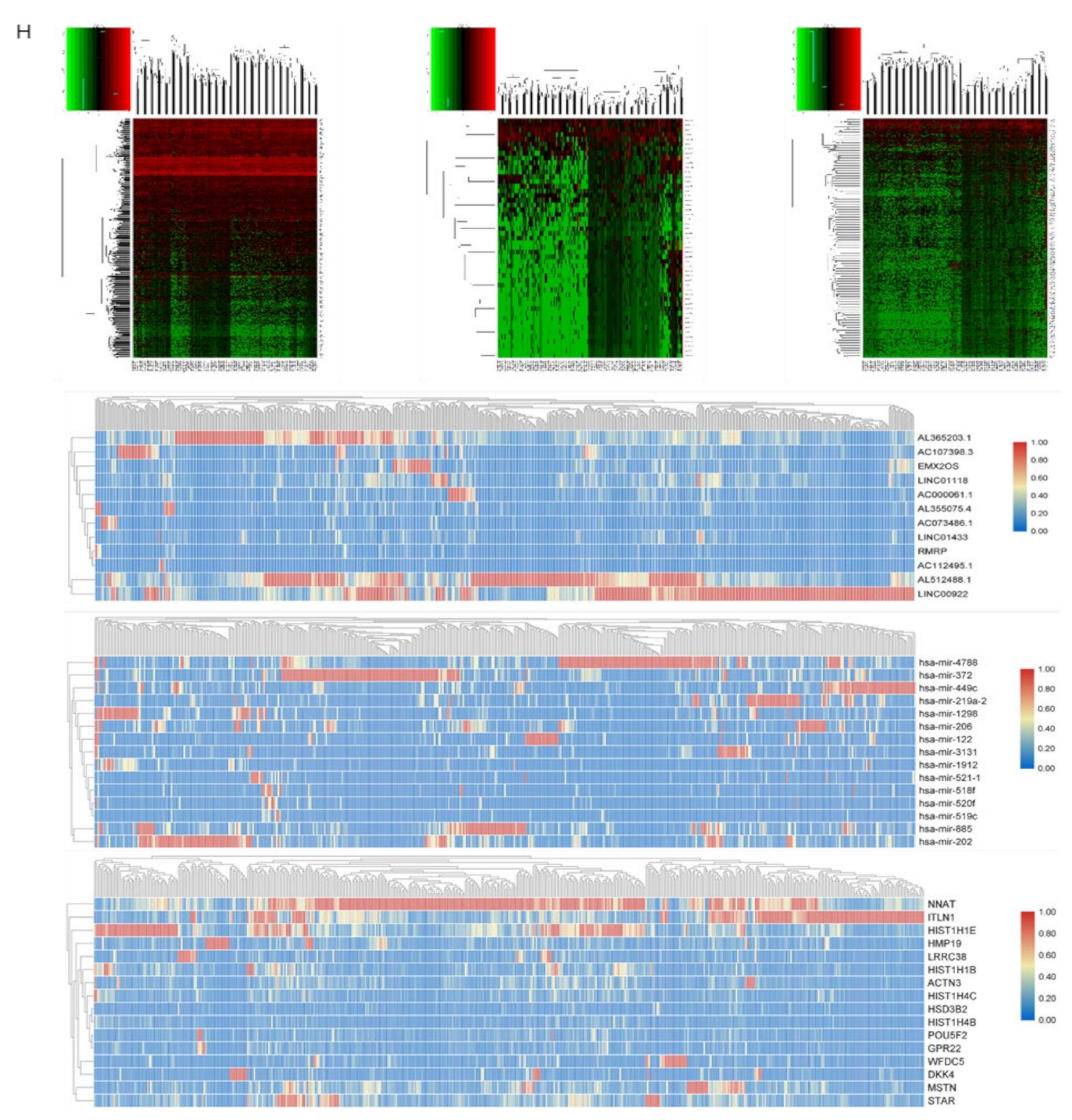
Distributions of expressed genes in LUAD (log2FC > 0.5 and adjusted *p*-value < 0.05). The volcano plot of 1292 differentially expressed long noncoding RNAs (DElncRNAs) (**A**), 147 differentially expressed microRNAs (DEmiRNAs) (**B**), and 1640 differentially expressed mRNAs (DEmRNAs) (**C**). Red stands for upregulation, and green stands for downregulation. The x-axis depicts the value of log2FC, and the y-axis depicts the value of log10 (*p*-value). In the competitive endogenous RNA (ceRNA) network constructed by differentially expressed genes related to MYC, green and blue represent downregulated genes, while red represents upregulated genes (**D**). The expression levels of WT1-AS, miR-206 and nicotinamide phosphoribosyltransferase (NAMPT) were significantly correlated with patient survival (**E**–**G**). Heat maps of differential genes (**H**).

The R software “edgeR” package was used to identify 468 differentially expressed lncRNAs; 257 (54.56%) were upregulated DElncRNAs, 214 (45.44%) were downregulated DElncRNAs, and the scatter plot (Figure 2A) and heat map (Figure 2H) of differentially expressed lncRNAs were drawn.

#### 3.2.2. Identification of DemiRNA in LUAD 

The R software edgeR package was used to identify 51 differentially expressed miRNA; the upregulated DEmiRNA was 15 (29.41%), the downregulated DEmiRNA was 36 (70.59%), and the scatter plot (Figure 2B) and heat map (Figure 2H) of the differentially expressed miRNA were drawn.

#### 3.2.3. Identification of DemRNA in LUAD 

The edgeR package of R software was used to identify 1640 differentially expressed mRNAs; the upregulated DEmRNA was 946 (57.68%), the downregulated DEmRNA was 694 (42.32%), and the scatter plot (Figure 2C) and heat map (Figure 2H) of differentially expressed mRNAs were drawn.

### 3.3. Prediction of Target 

#### 3.3.1. Prediction of Target miRNAs of DElncRNAs

LncRNA–miRNA targeting DEmiRNA was predicted through the miRcode database, and 1361 lncRNA–miRNA interactions combined with DElncRNA and DEmiRNA were identified to obtain the intersection of lncRNA–miRNA interactions. Finally, 60 lncRNA–miRNA interactions were identified, including five miRNA and 29 lncRNAs. 

#### 3.3.2. Prediction of Target mRNAs of DEmiRNAs

The five miRNAs obtained in the above step were respectively predicted in the miRDB, miRTarBase, and TargetScan databases, and then, the targeted mRNAs predicted in the above three databases were intersected with the different expressed mRNAs to obtain the final 59 DEmRNA networks, including five miRNAs and 59 mRNAs, differential lncRNA survival analysis (Figure S2A), and differential miRNA survival analysis (Figure S2B).

### 3.4. Construction of ceRNA Network and Survival Analysis 

#### 3.4.1. Construction of ceRNA Network

The ceRNA regulatory network was drawn in Figure 2D using Cytoscape software, including 30 lncRNAs, 5 miRNAs, and 83 mRNA, which was to better analyze the role of DElncRNAs in LUAD and further construct the ceRNA network by looking for the molecular mechanism of ceRNA regulation on LUAD.

#### 3.4.2. Survival Analysis for RNAs in the ceRNA Network

In order to study the effect of differentially expressed lncRNA, miRNA, and mRNA in the ceRNA network on the overall survival of patients with prognosis, the Kaplan–Meier univariate analysis was performed to analyze the effects on the survival of patients with LUAD. High WT1-AS (Figure 2E) and nicotinamide phosphoribosyltransferase (NAMPT) (Figure 2F) expressions were associated with a poor prognosis in LUAD patients, and low miR-206 (Figure 2G) expression was associated with poor prognosis in patients with LUAD. Differentially expressed lncRNA (Figure S3A), miRNA (Figure S3B), mRNA (Figure S3C) and correlation analysis of differentially expressed genes (Figure S4) were shown in sup-plementary material.

### 3.5. LncRNA WT1-AS and miRNA miR-206 Affects the Occurrence of LUAD

#### 3.5.1. The Expression Level of lncRNA WT1-AS in Tumor Cell Lines

**Figure 3:**
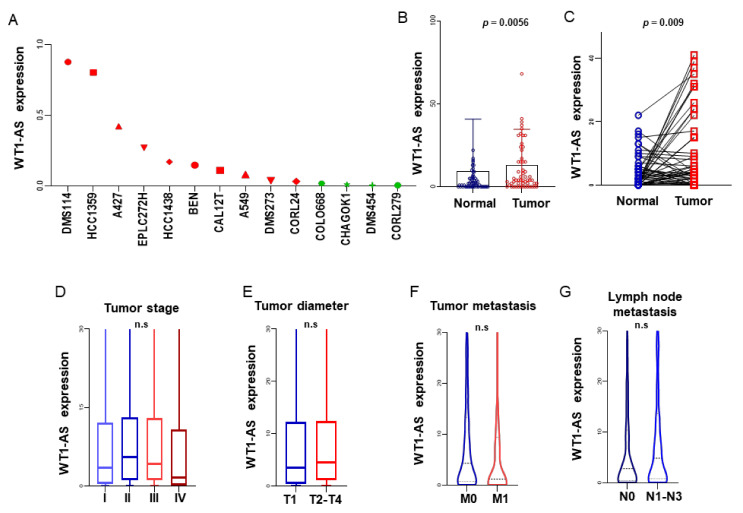
The expression levels of WT1-AS in different lung cancer cell lines were downloaded from CCLE (**A**). The expression level of WT1-AS in tumor tissues and adjacent tissues of LUAD patients was significantly different (**B**). Paired difference analysis (**C**). The relationship between WT1-AS and LUAD stage, tumor diameter, tumor metastasis, and lymph node metastasis (**D**–**G**).

The gene expression data of all tumor cell lines downloaded from the Cancer Cell Line Encyclopedia (CCLE) database and the comparative analysis found that the expression of WT1-AS in all LUAD cell lines was higher than that of other tumor cell lines, suggesting that the high expression of WT1-AS may influence the occurrence of lung cancer (Figure 3A). 

#### 3.5.2. Expression of WT1-AS between LUAD and Normal Lung Tissues

WT1-AS expression levels in 515 LUAD patients downloaded from the TCGA database. There were significant differences in the WT1-AS expression between tumor tissues and adjacent paracancer tissues in 59 patients, and the paired difference analysis results were consistent (*p* = 0.002; Figure 3B,C). The high expression level of MYC gene corresponds to the increased expression level of WT1-AS (*p* < 0.001). At the same time, the correlation between clinical features and the expression level of WT1-AS was analyzed by a single factor analysis, and the results showed that WT1-AS could affect the prognosis of patients as an independent indicator.

#### 3.5.3. Expression Relationship between WT1-AS and mi-R206

**Figure 4:**
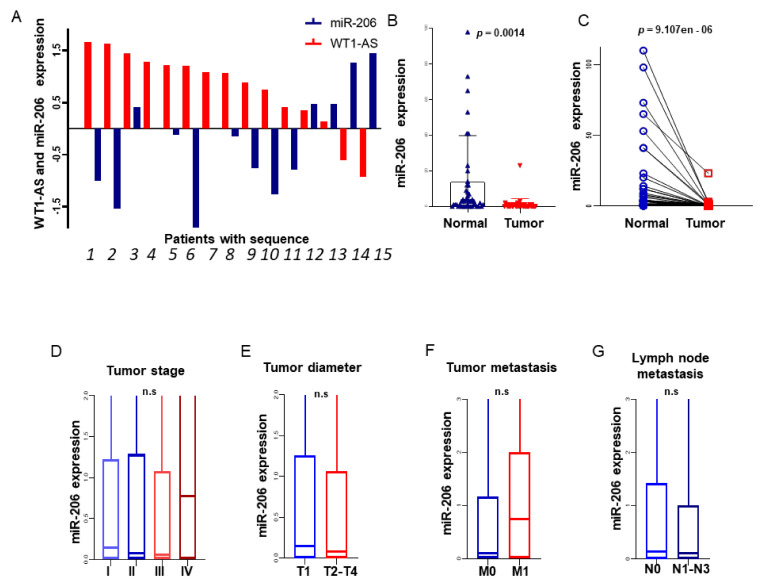
There was a negative correlation between the WT1-AS and miR-206 expression levels in the same patient (**A**). The expression level of miR-206 in tumor tissues and adjacent tissues of LUAD patients was significantly different (**B**). Paired difference analysis (**C**). The relationship between miR-206 and LUAD stage, tumor diameter, tumor metastasis, and lymph node metastasis (**D**–**G**).

The expression relationship shown in Figure 4A between WT1-AS and mi-R206 with LUAD and WT1-AS and miR-206 were significantly negatively regulated.

#### 3.5.4. Expression of miR-206 between LUAD and Normal Lung Tissues

The expression of miR-206 in 515 LUAD patients downloaded from the TCGA database. In the tumor tissues and adjacent paracancer tissues of 59 patients, miR-206 was significantly downregulated in the tumor samples, and the paired difference analysis results were consistent (*p* = 0.002; Figure 4B,C). The high expression level of the MYC gene corresponds to the decreased expression level of miR-206 (*p* < 0.001). At the same time, a correlation between the clinical features and miR-206 expression level was detected using a univariate analysis, and the results showed that miR-206 could affect the prognosis of patients as an independent indicator. Interestingly, we found a significant correlation between a smoking history and miR-206 expression levels in patients (Table 2). 

#### 3.5.5. The Expression Level of mRNA NAMPT in Tumor Cell Lines

**Figure 5:**
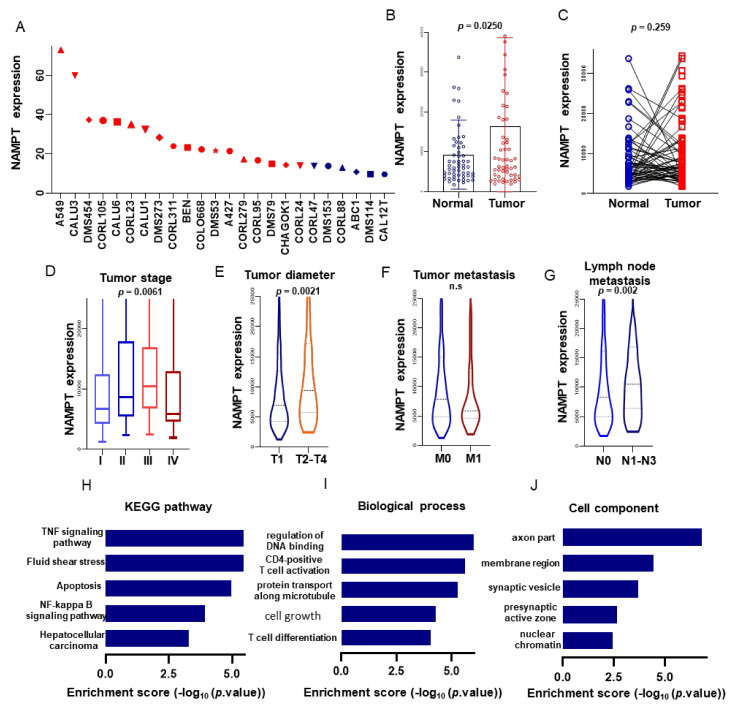
The expression levels of NAMPT in different lung cancer cell lines were downloaded from CCLE (**A**). The expression level of NAMPT in tumor tissues and adjacent tissues of LUAD patients was significantly different (**B**). Paired difference analysis (**C**). The relationship between NAMPT and LUAD stage, tumor diameter, tumor metastasis, and lymph node metastasis (**D**–**G**). KEGG analysis and GO analysis of differentially expressed genes related to NAMPT (**H**–**J**).

By comparing and analyzing the gene expression data of all tumor cell lines downloaded from the CCLE database, the expression level of NAMPT in all lung cancer cell lines was higher than that of other tumor cell lines, suggesting that NAMPT expression may be involved in the pathogenesis of lung cancer (Figure 5A).

### 3.6. mRNA NAMPT Affects the Occurrence of LUAD

#### 3.6.1. Expression of NAMPT between LUAD and Normal Lung Tissues

The WT1-AS expression levels in 515 LUAD patients were downloaded from the TCGA database. The NAMPT expression was significantly different in tumor tissues and adjacent tissues in 59 patients, and the paired difference analysis results were consistent (*p* = 0.002; Figure 5B,C). The high expression level of the MYC gene corresponds to the increased expression level of NAMPT (*p* < 0.001). At the same time, the association between clinical features and NAMPT expression level was analyzed for a single factor analysis, and the results showed that NAMPT could affect the prognosis of patients as an independent indicator. Moreover, a univariate analysis of clinical information showed that the NAMPT expression was significantly correlated with age, gender, tumor grade, lymph node metastasis, and tumor diameter. The smoking history and tumor metastasis of patients were not correlated with NAMPT (Table 2). 

#### 3.6.2. KEGG Analysis for NAMPT

The NAMPT gene of the KEGG analysis shown in Figure 5H, which was to explore the key regulatory pathways involved in NAMPT and use a co-expression analysis to search for NAMPT-related genes and perform a KEGG analysis. The KEGG results focused on the TNF signaling pathway, fluid shear stress, apoptosis, NF-kappa B signaling pathway, hepatocellular carcinoma, and other regulatory pathways; prompt NAMPT gene were associated with tumorigenesis.

#### 3.6.3. GO Analysis for NAMPT

The 299 genes co-expressed with NAMPT were analyzed for the GO analysis to detect the functional bias of the differentially expressed transcripts of these genes, which was to clarify the relationship between the differential expression pattern of the NAMPT gene in normal and tumor cells. The biological process analysis showed that the genes co-expressed with KEGG were enriched in the regulation of DNA binding, CD4-positive T-cell activation, protein transport along the microtubule, cell growth, and T-cell differentiation, which were closely related to tumor cell proliferation. The cell component analysis results indicated that co-expressed genes are enriched in the axon part, membrane region, synaptic vesicle, presynaptic active zone, and nuclear chromatin (Figure 5I,J).

### 3.7. ceRNA Network in LUSC

The gene expression data were downloaded from LUSC patients from the TCGA database. Interestingly, the analysis results showed that WT1-AS, miR-206, and NAMPT showed no significant differences in gene expression in LUSC patients. According to the expression of high and low group survival curve drawings, prompt differential expression of WT1-AS, miR-206, and NAMPT impacts were not significant on patient survival. 

## 4. Discussions

In recent years, more and more evidence has shown that the mutual regulation mode is closely correlated with the occurrence and development of tumors between lncRNA and miRNA and their downstream target genes, and it has become a focus point topic in the field of tumors. As a significant factor in post-transcriptional horizontal regulation, miRNA activity can be regulated by lncRNA through sponge adsorption, and lncRNA competitively binds to miRNA as ceRNA to regulate the protein level of coding genes and participate in regulating the biological behavior of cells. However, lncRNA that plays the role of ceRNA in tumors is still poorly understood [12,13,14]. In theory, any RNA species with miRNA-binding sites can bind to miRNAs, thus acting in the mode of ceRNA. Various molecules in the ceRNA network are in a certain balanced state under a normal physiological state. Once the balance is broken, it will lead to the occurrence of the disease. MiRNAs play a central role in the ceRNA regulatory network and play a negative regulatory role by binding to target mRNA [12,13,14].

Several lncRNAs have been found to control individual gene expression profiles through epigenetic modification or regulation transcription mechanisms. It has been proven that lncRNA could regulate the transcription of downstream genes, the mRNA shearing mode, and serve as the transcription prerequisite for small rRNAs [15]. The abnormal expression of lncRNA will affect the biological function of protein and lead to the disorder of the pathological process, thus participating in the pathogenesis of the disease. Harrison et al. found a small number of conserved lncRNA sequences through bioinformatics combined with a genomic sequence analysis, which are supposed to play an important role in the process of tumorigenesis [16]. Some researchers have confirmed that there are significant differences in lncRNA expression in cancer cells relative to paracarcinoma tissue samples from the uniform source [17]. Due to the heterogeneity of tumor samples, adenocarcinoma and squamous cell cancer cells may be present in the same tumor sample. Adenosquamous carcinoma is often erroneously diagnosed as LUAD or LUSC [18,19,20]. Recently, it has been reported in the literature that there are many differentially expressed lncRNAs in LUAD and LUSC, suggesting that lncRNA may be a potential biomarker for the diagnosis of NSCLC [21].

The MYC gene has been confirmed to be a key oncogene affecting many malignant tumors [22]. Many related studies have reported that MYC plays an important role in the development of LUAD. Interestingly, the difference in the expression level of the MYC gene was not reflected in the cancerous tissues and adjacent tissues of patients, and the differential expression of the MYC gene did not have a statistically significant effect on patients [23]. It was speculated that the MYC gene should play a role in tumor progression, and inhibiting the expression of MYC gene and changing its regulatory pathway may provide ideas for inhibiting tumor proliferation and metastasis. Our analysis showed that the highly malignant stages of LUAD correspond to higher MYC expression levels (*p* = 0.0276), and a high MYC expression is associated with LUAD tumor metastasis and a larger tumor diameter (*p* = 0.0225). The overexpression of the MYC gene has a negative impact on the tumor stage, tumor metastasis, lymph node metastasis, tumor diameter enlargement, and other poor prognosis factors of patients. This suggests that the MYC gene can be used as a prognostic diagnostic indicator, indicating the prognostic risk. Therefore, our research is based on the fact that MYC is a recognized key cancer gene [23]. The patient’s RNA sequencing data is divided into two groups with high expression and low expression, according to the median of the expression level of MYC. A ceRNA network was constructed significantly related to survival genes, which will provide support for subsequent cell experiments, molecular biology experiments, and in vitro experiments [24].

Long noncoding RNAs (lncRNAs) are a novel class of noncoding RNAs that are capable of regulating gene expressions at various levels. Recent scientific works have shown that lncRNAs regulate cell proliferation, apoptosis, autophagy, epithelial–mesenchymal transition (EMT), invasion, and metastasis of cancer by modulating gene expression and cancer-related signaling pathways, thus contributing to the onset and progression of cancer [25,26,27,28,29,30]. Researchers have reported the effects of WT1-AS on oxidative stress injuries (OSI) and the apoptosis of Alzheimer’s disease (AD) neurons and its specific correlation with the miR-375/SIX4 axis and WT1 expression mechanism. The overexpression of WT1-AS can inhibit the miR-375/SIX4 axis, OSI, and neuronal apoptosis in AD by inhibiting the expression of WT1 [29]. In addition, Qiu G et al. reported that the abnormal expression of WT1-AS leads to excessive activation of AKT. This deformity may lead to changes in the protective immune response in malignant tumors. Targeting WT1-AS, miR-494-3p, and AKT may be a new option for the treatment of glioma [30]. Our analysis results showed that the expression level of lncRNA WT1-AS in the MYC high expression group was significantly higher than that in the MYC low expression group, showing an upregulation trend. In the cancer tissues of patients, there was also a significant up-regulation trend compared with the adjacent tissues. In addition, combined with the patient’s clinical information, a high expression of WT1-AS indicates that the survival rate of patients is reduced, and it indicates the risk of poor prognosis. There is no significant statistical significance between WT1-AS and the patient’s age, gender, smoking history, tumor stage, tumor metastasis, lymph node metastasis, tumor diameter, etc. (*p* > 0.05).

MiRNA is a small, endogenous noncoding single-stranded RNA molecule that can target the target mRNA and cause the expression of the corresponding gene to be silenced, which regulates the generation and development of a variety of tumors, including lung cancer [31,32,33,34,35]. X Chen’s research reported that miR-206 is lowly expressed in LUAD tissues, and miR-206 in LUAD cells partially inhibits cell proliferation and migration by targeting the expression of the MET gene [35]. The results of this study showed that the expression of miR-206 in LUAD tissue was significantly lower than that in noncancerous lung tissue. Transfection upregulates the expression of miR-206 and can significantly inhibit the proliferation and migration of LUAD cells [32,35]. Our analysis data showed that miR-206 was not significantly correlated with patient age, gender, tumor stage, tumor metastasis, lymph node metastasis, and tumor diameter. However, the smoking history of patients was significantly correlated with the level of miR-206 (*p* = 0.011), suggesting that the connection between smoking and LUAD may be due to the influence of miR-206 as a tumor suppressor gene.

Nicotinamide phosphoribosyltransferase (NAMPT) is regarded as an important target for tumor treatment, and many studies have confirmed that NAMPT inhibitors have obvious antitumor effects [36,37,38,39,40]. NAMPT can directly regulate cell metabolism by controlling the synthesis of nicotinamide adenine dinucleotide (NAD), and it can also indirectly regulate cells by affecting the activity of NAD-dependent enzymes and upregulating the level of reduced nicotinamide adenine dinucleotide phosphate (NADPH) metabolism to improve cell viability [36,37,38]. The results of Audrito V proved that the nicotinamide adenine dinucleotide (NAD) biosynthetic enzyme NAMPT is the driving factor for the development ofB-Raf Proto-Oncogene, Serine/Threonine Kinase (B-RAF) resistance; cells overexpressing NAMPT turned into an aggressive/mesenchymal phenotype, upregulating the expression of Zinc Finger E-Box Binding Homeobox 1(ZEB1) and Twist Family BHLH Transcription Factor 1(TWIST1), two transcription factors that drive epithelial cells to mesenchymal transition (EMT) [40]. Our analysis results showed that NAMPT has a high expression trend in the patients with a high MYC expression group, and compared with adjacent tissues, NAMPT has a significant upregulation trend in patient tumor tissues. The results of the clinical analysis showed that the five-year survival rate of patients with a high expression of NAMPT-linked patients was reduced, indicating a prognostic risk. The expression level of NAMPT is related to the patient’s age, gender (highly expressed in women), tumor stage, tumor lymph node metastasis, and tumor diameter, while the expression level of NAMPT is not statistically related to the patient’s smoking history and tumor metastasis.

## 5. Conclusions

In conclusion, we established a ceRNA (WT1-AS/miR-206/NAMPT) expressed network related to the prognosis of LUAD, which is better for knowing the correlation among lncRNA–miRNA–mRNA. Additionally, we identified that the ceRNA-based WT1-AS/miR-206/NAMPT axis can be a novel significant prognostic factor participating in LUAD, and the prognostic model is useful for exploring the pathogenesis of LUAD.

## Figures and Tables

**Table 1:** Univariate analysis of the overall survival in lung adenocarcinoma (LUAD) patients based on clinical feature stratification and the relationship between MYC expressions.

Factor	Variable	*N*	*MYC* Expression (Median)	*p*-Value	Overall Survival			
					Years	Hazard Ration	95% CI	*p*-Value
					(Mean)	(Log-Rank Test)		(Log-Rank Test)
Age (diagnosis)								
≥60		354	2760.51	0.459	3.778	0.956	0.688 to 1.327	0.788
<60		133	2563.26		3.984	1.046	0.754 to 1.453	
Gender								
Male		232	2333.16	0.196	3.984	0.984	0.734 to 1.319	0.914
Female		265	2820.00		4.444	1.016	0.758 to 1.362	
Smoking history								
Never		71	1949.72	0.007	3.893	1.142	0.742 to 1.757	0.525
Positive		408	2760.81		4.186	0.876	0.569 to 1.347	
Tumor stage								
I		268	2410.16	0.036	7.178	0.348	0.257 to 0.4711	<0.0001
II–IV		222	2767.32		2.726	2.873	2.124 to 3.886	
Tumor metastasis								
none		330	2492.69	0.130	4.153	0.446	0.210 to 0.948	0.002
metastasis		23	3410.23		2.263	2.243	1.055 to 4.771	
Lymph node metastasis								
none		320	2532.83	0.344	6.351	0.391	0.283 to 0.541	<0.0001
metastasis		165	2692.27		2.608	2.555	1.848 to 3.533	
Diameter								
≥3 cm		328	2295.41	0.019	6.351	0.613	0.450 to 0.836	0.010
<3 cm		166	2768.38		3.718	1.630	1.196 to 2.222	

**Table 2:** Univariate analysis of the overall survival in LUAD patients based on clinical feature stratification and the relationship between WT1-AS, miR-206, and nicotinamide phosphoribosyltransferase (NAMPT) expression.

			*WT1-AS*	*p*-Value	*miR-206*		*NAMPT*	
Factor	Variable	*N*	Expression		Expression	*p*-Value	Expression	*p*-Value
			(Median)		(Median)		(Median)	
Age								
	≥60	354	3.658	0.779	0.084	0.577	8692.641	0.049
	<60	133	3.822		0.125		7458.040	
Gender								
	Male	232	4.178	0.206	0.124	0.346	7572.449	0.033
	Female	265	3.425		0.104		7769.888	
Smoking								
	Never	71	2.959	0.434	0.657	0.011	5916.100	0.942
	Positive	408	3.676		0.094		8378.513	
Tumor stage								
	I–II	268	3.662	0.911	0.119	0.750	7210.478	0.029
	III–IV	222	3.841		0.076		9495.610	
Metastasis								
	none	330	4.291	0.487	0.107	0.783	7873.821	0.425
	metastasis	23	1.217		0.740		5875.075	
Lymph node metastasis								
	none	320	3.490	0.605	0.144	0.507	6964.102	0.002
	metastasis	165	4.529		0.080		9377.424	
Diameter								
	≥3 cm	328	4.893	0.413	0.107	0.544	8328.164	<0.001
	<3 cm	166	2.834		0.145		6991.640	

## Data Availability

The data that support the findings of this study are available in the TCGA database at https://CancerGenome.nih.gov/. These data were derived from the following resources available in the public domain: https://portal.gdc.cancer.gov/.

## Supplementary Materials

The following are available online at https://www.mdpi.com/2218-273X/11/2/203/s1: Figure S1: Immunohistochemistry in patients with lung adenocarcinoma (MYC gene); Figure S2: Differential lncRNA survival analysis (A), differential miRNA survival analysis (B); Figure S3: Gene expression levels in paracancer tissues and cancer tissues of patients with differentially expressed genes (A) lncRNA, (B) miRNA, (C) mRNA; Figure S4: Correlation analysis of differentially expressed genes.
