# Supplementary material for: Unravelling the Role of LncRNA WT1-AS/miR-206/NAMPT Axis as Prognostic Biomarkers in Lung Adenocarcinoma
Source: Biomolecules. 2021 Feb 2;11(2):203. doi: 10.3390/biom11020203 (PMC7912827; doi:10.3390/biom11020203)
Supplement: Supplementary file 1 [file biomolecules-11-00203-s001.pdf]

Article

Supplementary materials: **Unravelling the Role of LncRNA WT1-AS/miR-206/ NAMPT Axis as Prognostic Biomarkers in Lung Adenocarcinoma**

Wen Li [1, 2†], Yu Liu [1†], Zi Jin Li [2], Yi Shi [1†], Jing Deng [2*], Jie Bai [2], Liang Ma [1], Xiao Xi Zeng [1], Shan Shan Feng [1], Jiali Ren [2], Feijun Luo[2], Duo Yan Rong [1], Xiao Qi Chen [2], Hua Qun Yin [3] , Zhu Chen [1*], Fu Da [2, 4*]

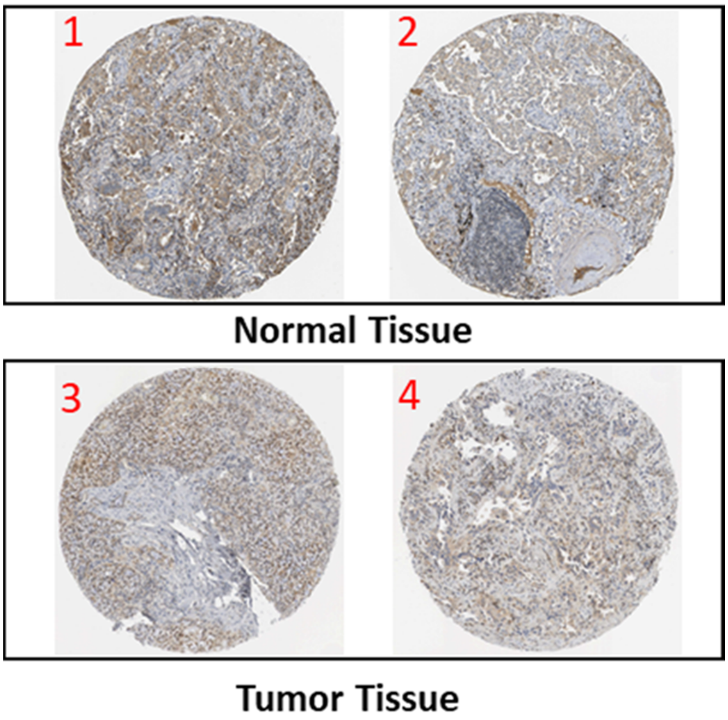

S.Fig.1 Immunohistochemistry in patients with lung adenocarcinoma (MYC gene)

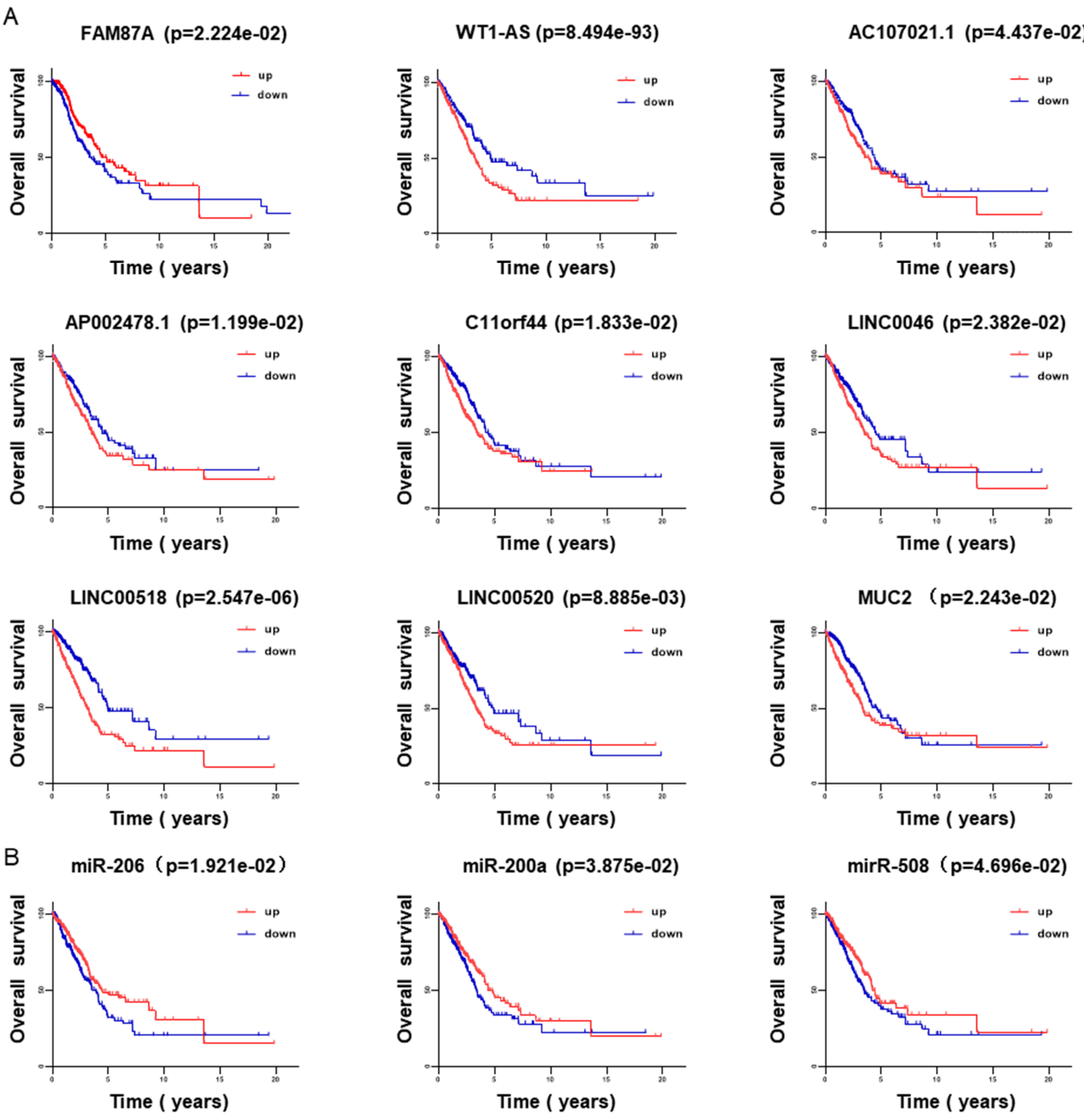

S.Fig.2 Differential lncRNA survival analysis (A), differential miRNA survival analysis(B)

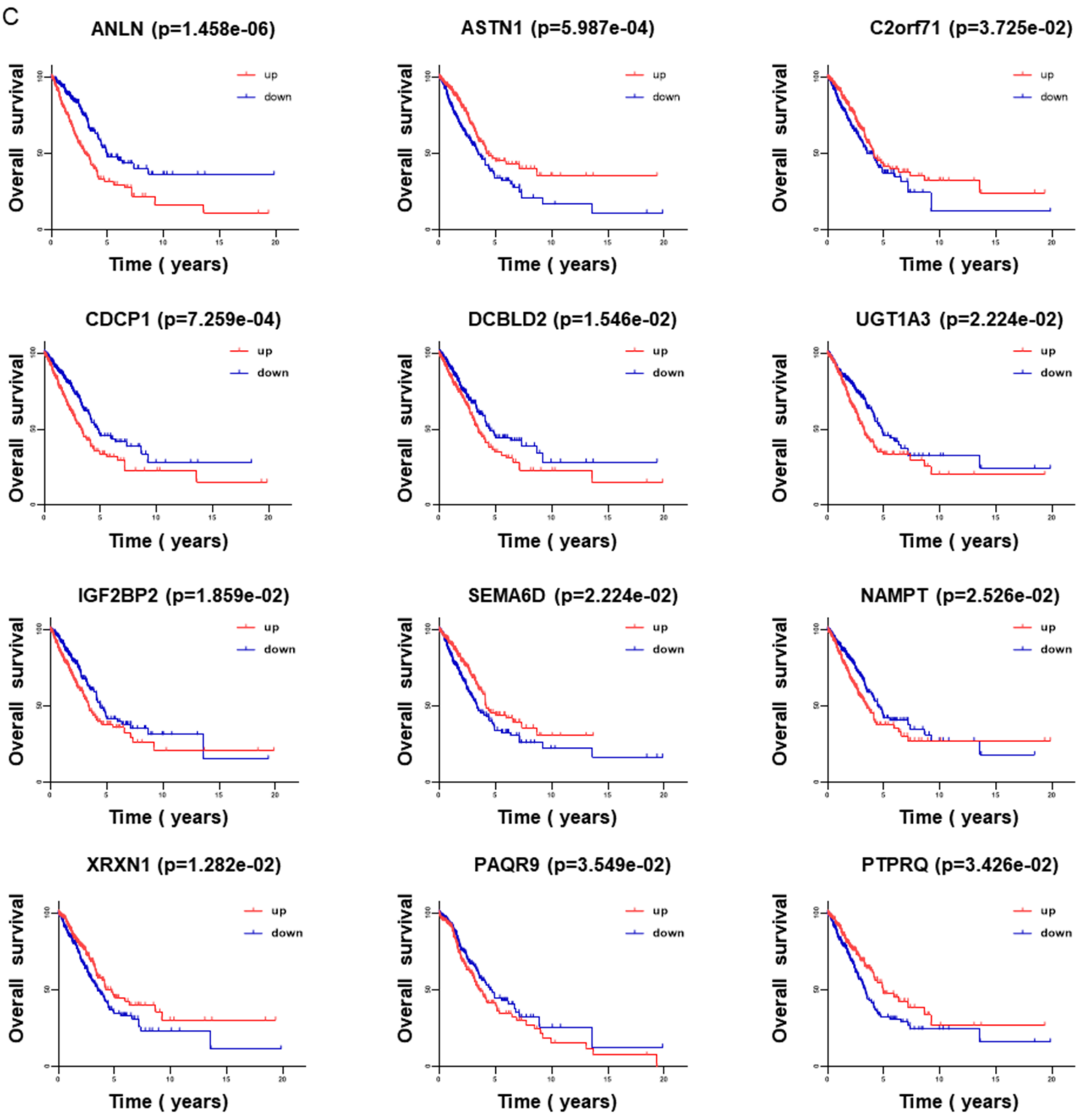

S.Fig.2 Differential mRNA survival analysis (C)

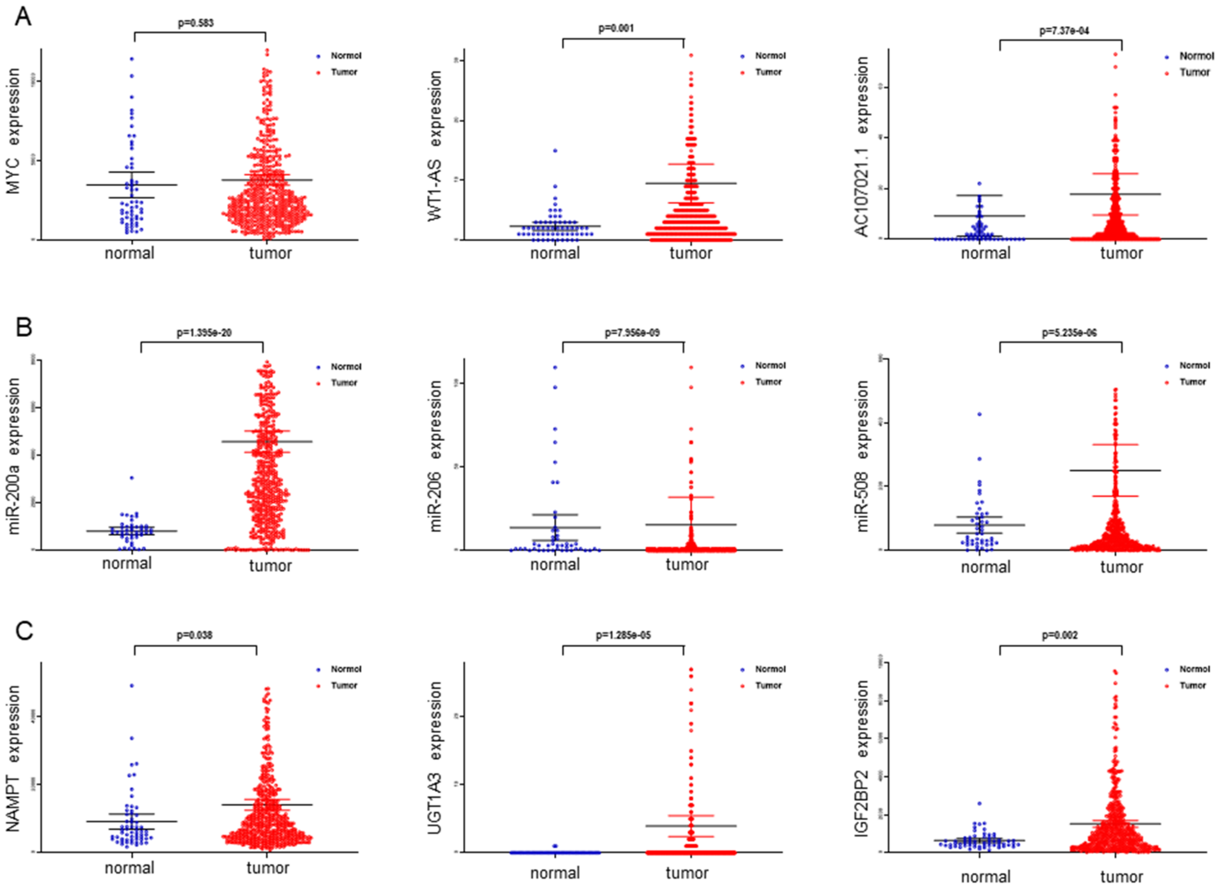

S.Fig.3 Gene expression levels in paracancer tissues and cancer tissues of patients with differentially expressed genes (A) lncRNA, (B) miRNA, (C) mRNA

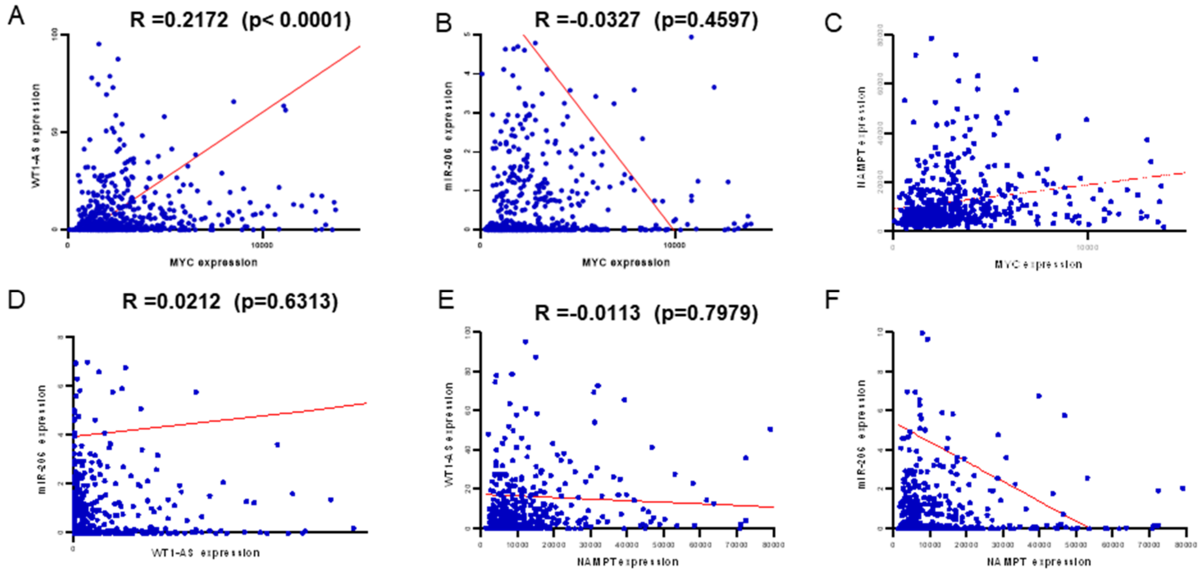

S.Fig.4 Correlation analysis of differentially expressed genes
